# Associations between diagnostic activity and measures of patient experience in primary care: a cross-sectional ecological study of English general practices

**DOI:** 10.3399/bjgp17X694097

**Published:** 2017-12-19

**Authors:** Georgios Lyratzopoulos, Silvia C Mendonca, Carolynn Gildea, Sean McPhail, Michael D Peake, Greg Rubin, Hardeep Singh, William Hamilton, Fiona M Walter, Martin Roland, Gary A Abel

**Affiliations:** Epidemiology of Cancer Healthcare and Outcomes (ECHO) Research Group, Department of Behavioural Science and Health, University College London, London, UK; Cambridge Centre for Health Services Research, Department of Public Health and Primary Care, Institute of Public Health, University of Cambridge, Cambridge, UK.; Cambridge Centre for Health Services Research, Department of Public Health and Primary Care, Institute of Public Health, University of Cambridge, Cambridge, UK.; National Cancer Registration and Analysis Services, Public Health England, London, UK.; National Cancer Registration and Analysis Services, Public Health England, London, UK.; National Cancer Registration and Analysis Services, Public Health England, London, UK; Institute for Lung Health, Department of Respiratory Medicine, University of Leicester, Leicester, UK.; Institute of Health and Society, Newcastle University, UK.; Houston Veterans Affairs Center for Innovations in Quality, Effectiveness and Safety, Michael E DeBakey Veterans Affairs Medical Center and the Section of Health Services Research, Department of Medicine, Baylor College of Medicine, Houston, US.; University of Exeter, Exeter, UK.; Primary Care Unit;; Cambridge Centre for Health Services Research, Department of Public Health and Primary Care, Institute of Public Health, University of Cambridge, Cambridge, UK.; University of Exeter, Exeter, UK.

**Keywords:** cancer, diagnosis, endoscopy, investigations, primary care, referrals

## Abstract

**Background:**

Lower use of endoscopies and urgent referrals for suspected cancer has been linked to poorer outcomes for patients with cancer; it is important to examine potential predictors of variable use.

**Aim:**

To examine the associations between general practice measures of patient experience and practice use of endoscopies or urgent referrals for suspected cancer.

**Design and setting:**

Cross-sectional ecological analysis in English general practices.

**Method:**

Data were taken from the GP Patient Survey and the Cancer Services Public Health Profiles. After adjustment for practice population characteristics, practice-level associations were examined between the use of endoscopy and urgent referrals for suspected cancer, and the ability to book an appointment (used as proxy for ease of access), the ability to see a preferred doctor (used as proxy for relational continuity), and doctor/nurse communication skills.

**Results:**

Taking into account practice scores for the ability to book an appointment, practices rated higher for the proxy measure of relational continuity used urgent referrals and endoscopies less often (for example, 30% lower urgent referral and 15% lower gastroscopy rates between practices in the 90th/10th centiles, respectively). In contrast, practices rated higher for doctor communication skills used urgent referrals and endoscopies more often (for example, 26% higher urgent referral and 17% higher gastroscopy rates between practices in the 90th/10th centiles, respectively). Patients with cancer in practices that were rated higher for doctor communication skills were less likely to be diagnosed as emergencies (1.7% lower between practices in the 90th than in the 10th centile).

**Conclusion:**

Practices where patients rated doctor communication highly were more likely to investigate and refer patients urgently but, in contrast, practices where patients could see their preferred doctor more readily were less likely to do so. This article discusses the possible implications of these findings for clinical practice.

## INTRODUCTION

Achieving accurate and timely diagnosis represents an important challenge for contemporary healthcare systems.^1^ In primary care, deciding appropriately whether a patient should be investigated or referred can be important for their outcome. Such decisions are traditionally seen as the doctor’s personal responsibility, but the influence of contextual factors is increasingly recognised.^2^^,^^3^

Although diagnostic delays occur in many conditions,^4^^,^^5^ cancer represents a useful disease model to study diagnostic delay.^6^^,^^7^ Cancer diagnosis often begins in primary care and involves decisions about investigations and referrals. Systematic underuse of investigations or referrals contributes to longer diagnostic intervals in patients with cancer, and has been linked to poorer cancer survival.^8^^–^10 Consequently, increasing attention is being paid to the role of primary care in cancer diagnosis.^11^^,^^12^

Previous evidence suggests positive practice-level associations between higher use of endoscopies or urgent referrals for suspected cancer (otherwise known as ‘2-week-wait’ referrals) and cancer outcomes including survival.^13^^,^^14^ Person-level data indicate that aspects of patient experience appear to be associated with the use of investigations or secondary care referrals; therefore, similar associations may also exist at the level of general practice.^15^^,^^16^ Understanding the associations between measures of patient experience and diagnostic activity in general practice can elucidate potential causes of variation, enabling the development of interventions.

All English general practices are rated for aspects of patient experience, including ease of access, ability to see a preferred doctor, and the quality of healthcare practitioner communication skills, using data from a large national patient survey — the GP Patient Survey.^17^ Another public reporting initiative — the Cancer Services Public Health Profiles — reports diagnostic activity indicators of relevance to cancer diagnosis in primary care.^18^^,^^19^ Against this background, this study aimed to examine whether general practice measures of patient experience are associated with higher or lower use of endoscopies or urgent referrals for suspected cancer.

**How this fits in**

Lower use of endoscopies and urgent referrals for suspected cancer in primary care has been linked to poorer outcomes for patients with cancer. Examining associations between these outcomes and measures of care experience, including proxy measures for access and continuity, may help to elucidate the mechanisms responsible for variation. In this study practices rated higher for a proxy measure of relational continuity used endoscopies and urgent referrals for suspected cancer less often. In contrast, practices rated higher for doctor communication used endoscopies and urgent referrals for suspected cancer more often, and had lower proportions of patients with cancer diagnosed as emergencies.

## METHOD

### Data

Thirteen indicators of diagnostic activity included in the 2013 release of the Cancer Services Public Health Profiles were used as the outcome measures in this study (Box 1). These included eight outcomes that, given evidence of their association with clinical outcomes in patients with cancer, were deemed of prime interest for our study, that is, the rates per 1000 registered patients of the use of gastrointestinal endoscopy and urgent referral for suspected cancer.^13^^,^^14^

Box 1. **Practice-level diagnostic activity indicators examined**

**Endoscopies or urgent referrals or suspected cancer (patients with/without cancer)**

Rates per 1000 registered practice patients:

- gastroscopy
- colonoscopy
- flexible sigmoidoscopy
- urgent referrals for suspected cancer (any site)
- urgent referrals for suspected colorectal cancer
- urgent referrals for suspected lung cancer
- urgent referrals for suspected skin cancer
- urgent referrals for suspected breast cancer

**Diagnostic outcome indicators (in patients with cancer)**

Proportion of all:

- urgent referrals for suspected cancer that resulted in cancer diagnosis (‘conversion rate’)
- treated patients with cancer whose diagnosis resulted from an urgent referral for suspected cancer (‘detection rate’)
- cancer diagnoses made after an emergency presentation
- cancer diagnoses made via a primary care referral
- cancer diagnoses made via ‘other’ diagnostic pathways

For the main exposure variables, data were used from the 2012/2013 GP Patient Survey, a questionnaire survey of patients registered with English general practices. In 2012/2013 there were more than 0.97 million responders (a 35% response rate). The study focused on five GP Patient Survey items because of evidence suggesting that they constitute important dimensions of patient satisfaction with primary care services.^20^ These measured the helpfulness of the practice receptionist; the ability to book appointments; the ability to see a preferred doctor (used as a proxy measure for continuity of care);^21^ doctor communication skills; and nurse communication skills (Appendix 1). Nurse communication skills were hypothesised a priori as unlikely to be associated with diagnostic activity.

Diagnostic activity indicator data related to 97% of all English general practices (excluding those with fewer than 1000 registered patients). Among the 7962 practices with diagnostic indicator data, eight had no GP Patient Survey scores, 692 had fewer than 100 GP Patient Survey responders, and 192 had missing deprivation values, leaving 7070 practices with complete data in the analysis sample.

### Analysis

To estimate the associations between diagnostic activity indicators and GP Patient Survey practice scores for each diagnostic activity indicator separately (treated as outcome variables), logistic or Poisson mixed-effect models were used (for proportion or rate indicators, respectively). GP Patient Survey standardised practice scores were included as the main exposure variables. The model also included random effect for practice (see below). Because patient experience scores are correlated across domains, practice scores were adjusted for all five items simultaneously (although ‘univariate’ associations are available from the authors on request).^20^^,^^22^^,^^23^ To adjust for the age–sex–deprivation profile of practice populations, all models additionally included 35 variables, each representing proportions of practice populations in specific age–sex strata; and the practice population deprivation quintile.^24^ Reported odds ratios or rate ratios represent how one standard deviation change in practice patient experience scores affects the odds or rates (as applicable) of a given diagnostic activity indicator.

As is customary, GP Patient Survey Likert scale items were converted to a 0–100 linear scale.^20^^,^^23^ The doctor and nurse communication skills items represent composite measures of five related sub-items, calculated as their mean (for responders answering at least three).^23^

Practice-level GP Patient Survey scores for each patient experience item represent shrunken estimates calculated from a linear regression model including a random effect for practice, adjusted for the sociodemographic characteristics of responders. Specifically, responder characteristics adjusted for in the calculation of practice GP Patient Survey scores included age (using nine age group categories, as included in the survey questionnaire), sex, ethnicity (white, mixed, Asian, black, Chinese, or other) and deprivation quintile (Indices of Multiple Deprivation 2010).^24^ Shrunken estimates of practice scores (otherwise known as best linear unbiased predictors) were used to reduce the effect of measurement error. The different distribution of practice scores for the five patient experience items would make comparisons of the effect sizes of the respective associations hard to interpret. Therefore, to enable comparisons practice scores were standardised, so that the resulting regression coefficients denote the change in the outcome associated with one standard deviation change in GP Patient Survey practice scores. This was achieved by dividing the estimated practice deviation from the national mean by the standard deviation of the random effect.

#### Population impact

To help appreciate the magnitude of associations in their natural (‘real life’) scale, the regression models were used to predict how higher/lower centile attainment of practice patient experience scores translates to practice differences in diagnostic process or outcome measures. Specifically, hypothetical scenarios were considered that assumed all practices in England attained the GP Patient Survey scores of certain centiles of the observed distribution (specifically, the 10th, 25th, 75th, and 90th centiles), and the levels of nationwide diagnostic process or outcome indicators corresponding to the 75th and 25th, or the 90th and 10th centiles of patient experience practice scores were subsequently compared. These illustrations assume that the observed associations are causal.

#### Sensitivity analysis

All main analyses were repeated after excluding 172 practices where more than 50% of GP Patient Survey responders had indicated that ‘there is usually only one GP in my GP surgery’ — indicating that these practices are mostly run as single-handed practices, rendering the proxy measure of relational continuity difficult to interpret.

## RESULTS

The analysis sample comprised 7070 practices. The mean number of registered patients in included practices was 7352 (range: 1012–46 126; standard deviation: 4236). The number of practices contributing data to each of the 13 indicators ranged from 6927 to 7070. The median general practice performance (positive experience) varied between 70% (for ability to see a preferred doctor) and 92% (for ability to book appointment).

Because the study sample was large, many statistically significant associations can be expected, which may, however, be of no practical importance. Consequently, hereafter the study focuses on significant associations with odds/rate ratio values ≤0.96 or ≥1.04 (for one standard deviation change). This was a post-hoc decision, motivated by the need to focus on the findings most likely to be of greater practical importance.

### Patient experience measures and use of endoscopies or urgent referrals for suspected cancer

Proxy measures of ease of access (helpfulness of receptionist and ability to book an appointment) and nurse communication skills were overall not associated with endoscopies or urgent referrals or suspected cancer (Table 1).

**Table 1.** Standardised coefficients for the association between practice-level GP Patient Survey scores and diagnostic activity indicators^a,b^

**Indicators**	**Q4: Helpful receptionists**		**Q12: Access to appointments**		**Q9: Ability to see a preferred doctor**		**Q21: Composite doctor communication**		**Q23: Composite nurse communication communication**	

	**OR/RR (95% CI)**	***P***	**OR/RR (95% CI)**	***P***	**OR/RR (95% CI)**	***P***	**OR/RR (95% CI)**	***P***	**OR/RR (95% CI)**	***P***
				
***Process indicators***										
Sigmoidoscopy rate	**0.96 (0.94 to 0.98)**	**<0.0001**	0.98 (0.96 to 0.99)	0.0087	0.97 (0.96 to 0.99)	0.0002	**1.05 (1.03 to 1.06)**	**<0.0001**	1.02 (1.01 to 1.04)	0.0065
Colonoscopy rate	1.00 (0.98 to 1.01)	0.8293	0.97 (0.96 to 0.99)	0.0002	0.98 (0.97 to 0.99)	0.0001	**1.04 (1.03 to 1.05)**	**<0.0001**	1.01 (1.00 to 1.02)	0.2031
Gastroscopy rate	0.99 (0.97 to 1.00)	0.0300	0.97 (0.96 to 0.98)	<0.0001	**0.94 (0.93 to 0.95)**	**<0.0001**	**1.07 (1.06 to 1.08)**	**<0.0001**	1.01 (1.00 to 1.03)	0.0183
Urgent referral^c^ rate	0.99 (0.97 to 1.00)	0.0704	0.99 (0.98 to 1.01)	0.3887	**0.86 (0.86 to 0.87)**	**<0.0001**	**1.11 (1.09 to 1.12)**	**<0.0001**	1.03 (1.01 to 1.04)	0.0001
Urgent referral^c^ rate (colorectal)	0.98 (0.96 to 0.99)	0.0082	1.00 (0.98 to 1.02)	0.8047	**0.86 (0.85 to 0.88)**	**<0.0001**	**1.12 (1.11 to 1.14)**	**<0.0001**	1.01 (1.00 to 1.03)	0.1408
Urgent referral^c^ rate (lung)	0.99 (0.97 to 1.02)	0.6580	0.98 (0.96 to 1.01)	0.1800	**0.92 (0.90 to 0.93)**	**<0.0001**	**1.08 (1.05 to 1.10)**	**<0.0001**	1.01 (0.98 to 1.03)	0.6400
Urgent referral^c^ rate (skin)	1.00 (0.97 to 1.02)	0.6808	0.98 (0.96 to 1.00)	0.0650	**0.87 (0.85 to 0.88)**	**<0.0001**	**1.08 (1.06 to 1.11)**	**<0.0001**	**1.04 (1.02 to 1.06)**	**0.0004**
Urgent referral^c^ rate (breast)	1.02 (1.00 to 1.03)	0.0697	0.99 (0.97 to 1.00)	0.1286	**0.91 (0.89 to 0.92)**	**<0.0001**	**1.04 (1.02 to 1.05)**	**<0.0001**	1.03 (1.02 to 1.05)	<0.0001

***Outcome indicators***										
Urgent referral^c^ conversion rate	1.00 (0.99 to 1.02)	0.5372	1.00 (0.99 to 1.02)	0.5526	**1.09 (1.07 to 1.10)**	**<0.0001**	**0.96 (0.95 to 0.98)**	**<0.0001**	1.01 (0.99 to 1.02)	0.4287
Urgent referral^c^ detection rate	1.01 (0.99 to 1.02)	0.5714	1.01 (0.99 to 1.03)	0.2695	**0.95 (0.94 to 0.97)**	**<0.0001**	**1.06 (1.05 to 1.08)**	**<0.0001**	0.99 (0.97 to 1.01)	0.3171
Emergency route to diagnosis	1.02 (1.00 to 1.04)	0.0312	0.98 (0.96 to 1.00)	0.0145	1.00 (0.99–1.02)	0.7575	**0.96 (0.94 to 0.98)**	**<0.0001**	1.00 (0.98 to 1.02)	0.9196
Referred route to diagnosis	0.98 (0.96 to 0.99)	0.0031	1.02 (1.01 to 1.04)	0.0096	0.97 (0.96–0.99)	0.0002	1.03 (1.02 to 1.05)	<0.0001	1.01 (1.00 to 1.03)	0.0879
Other route to diagnosis	1.01 (0.99 to 1.03)	0.2651	0.99 (0.97 to 1.02)	0.5776	1.03 (1.01–1.05)	0.0002	1.00 (0.98 to 1.01)	0.6434	0.98 (0.96 to 1.00)	0.0739

a *The results are from ‘multivariate’ models including practice scores for all five aspects of experience shown and adjusted by the age–sex–deprivation make-up of practice populations. The main exposure variables for GP Patient Survey items are standardised coefficients (shrunken* z *scores) instead of the observed practice scores.*

b *The associations highlighted in bold correspond to odds ratio/rate ratio values* ≥ *a 4% difference from parity (that is,* ≤*0.96 or* ≥*1.04).*

c *Urgent referral for suspected cancer is also known as ‘2-week wait’ referral. CI* = *confidence interval. OR* = *odds ratio. RR* = *rate ratio.*

**Figure 1.**
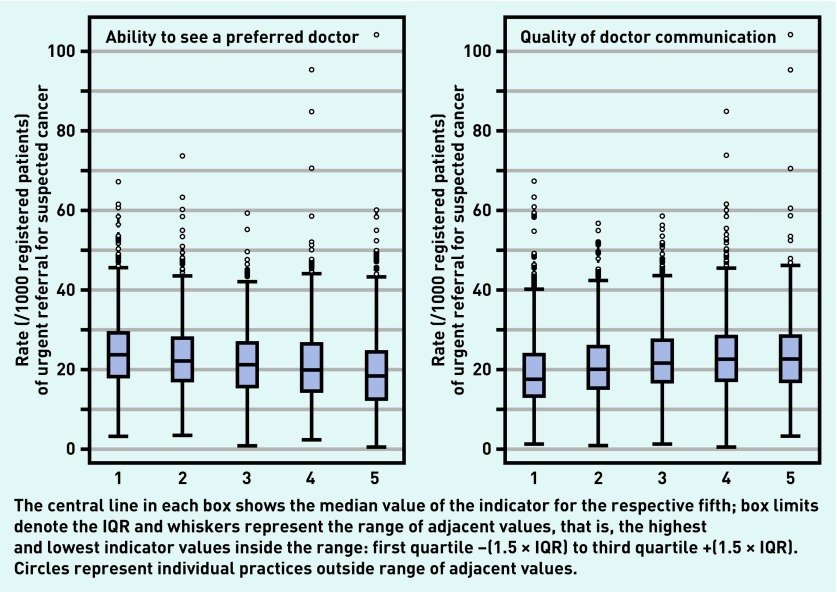
***Distribution of rates (per 1000 registered patients) of urgent referrals for suspected cancer, by fifth of practice scores for ability to see a preferred doctor (left) and doctor communication (right). IQR = interquartile range.***

**Figure 2.**
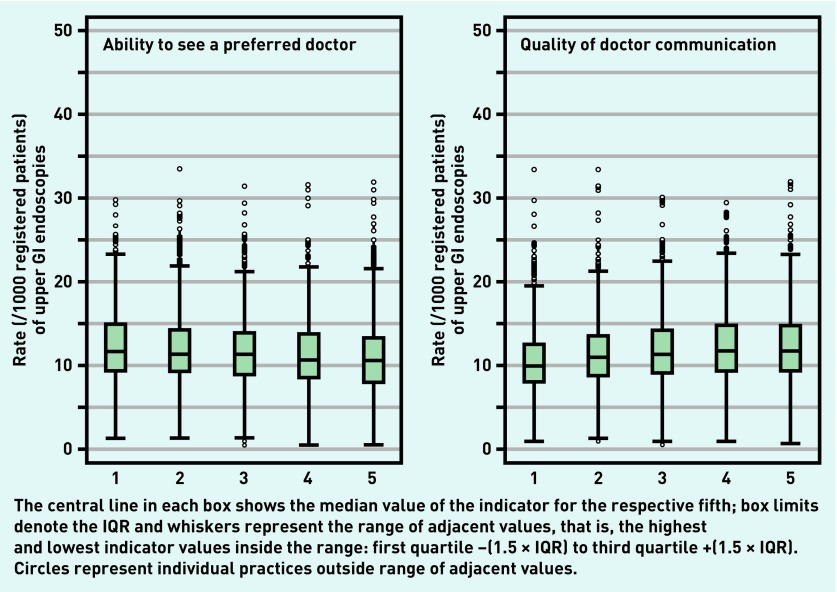
***Distribution of rates (per 1000 registered patients) of upper gastrointestinal endoscopies, by fifth of practice scores for ability to see a preferred doctor (left) and doctor communication (right). GI = gastrointestinal. IQR = interquartile range.***

In contrast, there were relatively strong associations between nearly all endoscopy or urgent referral outcomes and both the ability to see a preferred practice doctor and doctor communication skills (Figures 1 and 2). Practices with higher scores for ability to see a preferred doctor tended to have lower endoscopy and urgent referral rates. In contrast, practices with higher doctor communication scores tended to have higher endoscopy and urgent referral rates.

### Patient experience measures and diagnostic outcome indicators

As for endoscopy and urgent referrals, there were strong associations between the examined diagnostic outcome measures and both the ability to see a preferred doctor and doctor communication skills (Table 1). Specifically, practices with higher scores for ability to see a preferred doctor were more likely to have higher conversion rates (higher proportions of urgent referrals that resulted in a cancer diagnosis) and lower detection rates (lower proportions of patients with cancer detected following urgent referrals). The opposite associations were observed for practices rated higher for doctor communication skills. Higher practice scores for doctor communication skills were additionally associated with lower proportions of patients with cancer diagnosed after an emergency presentation.

### Illustration of impact (assuming causality)

In general, the impact is small when considering individual patients, reflecting that endoscopies and urgent referrals for suspected cancer are overall rare among patients registered with a practice; however, the impact is fairly large when considering relative change, which matters for service demand.

For the ability to see a preferred doctor, an increase in practice scores from the 25th to 75th centile would be associated with a decrease in urgent referral rates from 23.7 to 19.6 (per 1000 person-years), a relative difference of −17.4% (Table 2). Similarly, an increase in practice scores from the 10th to the 90th centile would be associated with a decrease from 26.0 to 18.2 (per 1000 person-years), a relative difference of −30.2%.

**Table 2.** Impact of changes in general practice scores for ability to see a preferred doctor (used as a proxy measure of relational continuity) and quality of doctor communication^a^

**Ability to see a preferred doctor**								

**Indicators**	**25th percentile**	**75th percentile**	**Difference (75th – 25th)**	**% Difference**	**10th percentile**	**90th percentile**	**Difference (90th – 10th)**	**% Difference**
***Process indicators***								
Gastroscopy rate	11.9	10.9	−1.0	−8.3	12.4	10.5	−1.9	−15.1
Urgent referral^b^ rate	23.7	19.6	−4.1	−17.4	26.0	18.2	−7.8	−30.2
Urgent referral^b^rate (colorectal)	4.1	3.4	−0.7	−17.7	4.5	3.1	−1.4	−30.7
Urgent referral^b^ rate (lung)	0.9	0.8	−0.1	−10.9	1.0	0.8	−0.2	−19.5
Urgent referral^b^ rate (skin)	4.0	3.4	−0.7	−17.0	4.4	3.1	−1.3	−29.6
Urgent referral^b^ rate (breast)	4.1	3.6	−0.5	−12.2	4.4	3.4	−0.9	−21.6

***Outcome indicators***								
Urgent referral^b^ conversion rate	9.7	10.6	1.0	10.1	9.2	11.1	1.8	19.9
Urgent referral^b^ detection rate	48.3	46.7	−1.6	−3.3	49.1	46.1	−3.0	−6.0

**Doctor communication**								

**Indicators**	**25th percentile**	**75th percentile**	**Difference (75th – 25th)**	**% Difference**	**10th percentile**	**90th percentile**	**Difference (90th – 10th)**	**% Difference**
***Process indicators***								
Sigmoidoscopy rate	4.5	4.7	0.2	5.3	4.4	4.8	0.5	11.1
Colonoscopy rate	6.8	7.1	0.3	4.6	6.6	7.2	0.6	9.4
Gastroscopy rate	11.1	12.0	0.9	8.1	10.6	12.4	1.8	17.0
Urgent referral^b^ rate	21.2	23.7	2.5	12.0	19.7	24.8	5.1	25.7
Urgent referral^b^ rate (colorectal)	3.7	4.2	0.5	14.0	3.4	4.4	1.0	30.4
Urgent referral^b^ rate (lung)	0.9	0.9	0.1	8.5	0.8	1.0	0.1	17.9
Urgent referral^b^ rate (skin)	3.6	4.0	0.3	9.5	3.5	4.1	0.7	20.1
Urgent referral^b^ rate (breast)	3.9	4.0	0.2	4.1	3.8	4.1	0.3	8.5

***Outcome indicators***								
Urgent referral^b^ conversion rate	10.1	9.8	−0.4	−3.7	10.4	9.6	−0.8	−7.3
Urgent referral^b^ detection rate	46.9	48.6	1.7	3.6	45.9	49.3	3.5	7.5
Emergency route to diagnosis	23.9	23.1	−0.8	−3.4	24.4	22.8	−1.7	−6.8

a Practices with the same age–sex–deprivation population make-up are compared at different centiles of the distribution of either outcome. Reported values are adjusted for the five outcomes shown in Table 1 and are on the relevant scale for each indicator (either rate or proportion). Only significant effect sizes >1.04 or <0.96 are visualised.

b Urgent referral for suspected cancer, otherwise also known as ‘2-week wait’ referrals.

For doctor communication skills, a 25th-to-75th centile increase in practice scores would be associated with an increase in urgent referral rates for suspected cancer from 21.2 to 23.7 (per 1000 person-years), a relative difference of +12.0% (Table 2); and a 10th-to-90th centile increase would be associated with an increase from 19.7 to 24.8 (per 1000 person-years), a relative difference of +25.7. Lastly, a 25th-to-75th centile increase in doctor communication skills scores would be associated with decreases from 23.9% to 23.1% in the proportion of patients with cancer diagnosed as emergencies, whereas for a 10th-to-90th centile increase the corresponding decrease would be from 24.4% to 22.8%.

### Sensitivity analysis

Excluding practices with more than 50% of responders indicating that ‘there is usually only one GP in my GP surgery’ produced practically identical findings to those observed in the main analysis (further details are available from the authors on request).

## DISCUSSION

### Summary

General practices that were rated highly for the ability to see a preferred doctor (used as a proxy for care continuity) tended to use endoscopies and urgent referrals for suspected cancer less frequently. The opposite was true for practices rated highly for doctor communication skills, which also had lower proportions of their patients with cancer diagnosed as emergencies. The size of these associations is small for an individual patient, but relatively large at the healthcare system level.

### Strengths and limitations

This study used nationwide data on objectively measured diagnostic activity and a well-characterised nationwide survey of patient experience. Appropriate modelling strategies were used to estimate associations, and have additionally provided evidence to help appreciate their size and impact.

The findings are limited, however, by the lack of individual-level data. This means that the actual experience of doctor communication of investigated or referred patients may differ from that of patients of the same practice who responded to the GP Patient Survey (an example of ecological fallacy). Although it was not possible to adjust for the individual characteristics of referred or investigated patients, adjustment for the age, sex, and deprivation profile of practice population could have provided for adequate adjustment.

Another limitation is the lack of adjustment for contextual confounders, such as the variable availability of direct access to endoscopy services among general practices. However, variable availability of direct-access endoscopy services is unlikely to be a strong confounder, as for that to occur there must be a high degree of co-clustering of both high/low provision of endoscopy and high/low experience scores in practices surrounding a hospital (endoscopy service), which is unlikely.

In the absence of a direct measure of continuity, a proxy measure was used (ability to see the GP of choice), as previously described.^20^ As the relationship between the ability to see the GP of choice and relational continuity will be imperfect (that is, some patients may have perfect continuity with a non-preferred doctor), the estimates of practice-level associations of diagnostic activity indicators with this measure of continuity may be conservative (underestimated).

The study’s measures of endoscopy use in general practice populations also include some endoscopies ordered by doctors other than the patient’s own GP (such as in secondary care settings).^14^

When patients rate GP communication during a consultation to be poor, trained professional assessors evaluating the video of the same consultation also tend to do the same, supporting the validity of patient surveys.^25^

Variable non-response to the GP Patient Survey presents a theoretical concern, but the degree of bias in organisational ratings that may result from differential non-response is small once adjustment has been made for patient case-mix, as in the current study.^26^

Last, the analysis excluded a few hundred practices, chiefly those with a small number of GP Patient Survey responses (<100); this limits the generalisability of the findings, because theoretically the associations between the studied measures of patient experience and diagnostic activity may differ in those (typically small) practices.

### Comparison with existing literature

There is little previous evidence of relevance. A study of 600 practices in an English region found that those practices rated higher for patients’ ability to see a preferred doctor had lower proportions of their patients with cancer diagnosed after urgent referrals for suspected cancer (that is, lower detection rates); further, the opposite was observed for practices rated higher for ‘confidence and trust’ in doctors.^27^ The current study amplifies this previous work substantially, by examining associations between measures of patient experience and 13 diagnostic activity indicators in a nationwide sample.

Another study examined associations between an index of care continuity (taking into account consultations with different clinicians up to 2 years before a cancer diagnosis) and diagnostic intervals for cancer, reporting weak and inconsistent associations across patients with three different cancers.^28^

### Implications for research and practice

Using cancer diagnosis as an exemplar, the findings indicate that both care continuity (measured as ability to see a preferred doctor) and doctor communication skills are associated with clinician decision making about diagnostic evaluation. It must be noted that, although evidence indicates that lower use of urgent referrals for suspected cancer and endoscopies is associated with poorer clinical outcomes in patients with cancer,^13^^,^^14^ higher rates will increase resource use and have the potential for psychological or even physical harm in some patients. Nonetheless, greater cancer-related diagnostic activity can also provide for earlier diagnosis of other serious (non-neoplastic) disease, and patients prefer to be investigated for symptoms of low predictive value for cancer.^29^^–^31 Although the 2015 National Institute for Health and Care Excellence guidelines for suspected cancer implicitly suggest that greater than historical levels of use of urgent referrals would be beneficial,^32^ the optimal level of use of urgent referrals and endoscopies cannot be determined by the findings of the current study, which, however, highlight potential mechanisms that lead to their higher or lower use.

Regarding potential mechanisms linking care continuity with lower use of referrals or investigations, there may be a tendency in practices with high levels of continuity for ‘new’ symptoms to be attributed to previous morbidity. There have been incidents where the diagnosis of lung cancer was missed because of chronic obstructive pulmonary disease comorbidity.^33^

Regarding potential mechanisms linking doctor communication skills with lower thresholds for investigating or referring a patient, three main hypotheses can be considered. First, doctors who are good communicators may obtain a more complete patient history, maximising the chances that their patients would meet investigation or referral criteria — a ‘mechanistic’ hypothesis. Second, patients may be more able to influence decisions about investigations or referrals when their doctors display greater empathy, consistent with evidence that most patients express preferences for investigation for suspected cancer at low levels of risk.^12^^,^^31^ Third, the observed associations may not be causal. For example, doctors who are good communicators may also inherently tend to use more investigations/referrals. ‘Reverse causality’ is also possible, given that the average patient is likely to be pro-investigation/pro-referral.^16^^,^^31^ If so, it might not be that a doctor’s better communication skills lead to more referrals and investigations, but that a doctor’s tendency to refer and investigate more leads them to be rated higher for the communication skills.

The findings have three main implications for research, policy, and clinical practice. First, although ease of access to primary care is an important dimension of patient experience and healthcare system quality, it reassuringly seems that, once a patient has been seen, these measures are not associated with how often endoscopies or urgent referrals for suspected cancer are used. Second, the possible association between investigation and referral and good communication would be consistent with careful listening to patients’ concerns being part of good clinical practice, potentially leading to more accurate diagnosis. However, although patients may well express a wish to be referred for new symptoms, this is not always in their best interests and may not be making the best use of NHS resources. The balance of risks and benefits of investigation and referral need to be discussed with patients; something that requires both time and good communication skills. It is possible that, regarding communication skills, the direction of causation is in the opposite direction — in other words, being investigated and referred leads patients to rate their GP more highly. Although this is possible, it is less likely because few of the random sample of practice patients who responded to the GP Patient Survey would have actually had symptoms requiring endoscopy or urgent referral. Third, continuity of care is widely seen as an important aspect of quality of care in general practice. Nevertheless, there are potential drawbacks, for example, overfamiliarity with patients’ complaints could lead GPs to be less inclined to investigate new symptoms, so doctors need to maintain a critical outlook on new symptoms.^34^ ‘Discontinuity’ may provide a ‘second opinion’ mechanism that can lead to faster diagnostic resolution.

## Appendix 1. Information on the GP Patient Survey items

**Table:**

The 2013 survey questionnaires can be accessed online (via https://gp-patient.co.uk/surveys-and-reports#december-2013). All items used in the study are reproduced verbatim below.
***Helpfulness of receptionist***
**Q4: How helpful do you find the receptionists at your GP surgery?** Very helpful; Fairly helpful; Not very helpful; Not at all helpful; Don’t know
***Ability to see a preferred doctor (used as a proxy measure of care continuity)***
**Q9: How often do you see or speak to the GP you prefer?** Always or almost always; A lot of the time; Some of the time; Never or almost never; Not tried at this GP surgery
[Filter Q8: Is there a particular GP you usually prefer to see or speak to? Yes — filtering to Q9 as above; No — Go to Q10; There is usually only one GP in my GP surgery — Go to Q10]
***Ability to book an appointment***
Q12: [Last time you wanted to see or speak to a GP or nurse from your GP surgery:] **Were you able to get an appointment to see or speak to someone?** Yes; Yes, but I had to call back closer to or on the day I wanted the appointment; No — Go to Q16; Can’t remember — Go to Q18
***Doctor communication skills^a^***
**Q21: Last time you saw or spoke to a GP from your GP surgery, how good was that GP at each of the following?**
**Giving you enough time:** Very good; Good; Neither good nor poor; Poor; Very poor; Doesn’t apply
**Listening to you:** Very good; Good; Neither good nor poor; Poor; Very poor; Doesn’t apply
**Explaining tests and treatments:** Very good; Good; Neither good nor poor; Poor; Very poor; Doesn’t apply
**Involving you in decisions about your care:** Very good; Good; Neither good nor poor; Poor; Very poor; Doesn’t apply
**Treating you with care and concern:** Very good; Good; Neither good nor poor; Poor; Very poor; Doesn’t apply
***Nurse communication skills^a^***
**Q23: Last time you saw or spoke to a nurse from your GP surgery, how good was that nurse at each of the following?** [Five sub-items identical to those used for doctor communication, above].

a Consistent with prior research, for the doctor and the nurse communication items, a composite was calculated as the mean of five sub-items when at least three of them had been answered.
